# The Interplay of PR Interval and AV Pacing Delays Used for Cardiac Resynchronization Therapy in Heart Failure Patients: Association with Clinical Response in a Retrospective Analysis of a Large Observational Study

**DOI:** 10.3390/jpm12091512

**Published:** 2022-09-15

**Authors:** Maurizio Gasparini, Mauro Biffi, Maurizio Landolina, Giuseppe Cattafi, Roberto Rordorf, Giovanni Luca Botto, Giovanni Battista Forleo, Giovanni Morani, Luca Santini, Antonio Dello Russo, Antonio Rossillo, Sarah Meloni, Andrea Grammatico, Marco Vitolo, Giuseppe Boriani

**Affiliations:** 1 Electrophysiology and Pacing Unit, Humanitas Clinical and Research Hospital, IRCCS, 20089 Rozzano, Italy; 2 Department of Experimental, Diagnostic and Specialty Medicine, S. Orsola-Malpighi University Hospital, University of Bologna, 40138 Bologna, Italy; 3 Cardiology Department, Maggiore Hospital, 26013 Crema, Italy; 4 Niguarda Hospital, 20162 Milano, Italy; 5 Cardiology Department, Policlinico San Matteo Pavia Fondazione, IRCCS, 27100 Pavia, Italy; 6 Cardiology Department, Ospedale di Rho, 20017 Rho, Italy; 7 Cardiology Department, Ospedale Luigi Sacco, 20157 Milano, Italy; 8 Cardiology Department, Ospedale Civile Maggiore, 37126 Verona, Italy; 9 Cardiology Department, Ospedale G.B. Grassi, 00122 Rome, Italy; 10 Cardiology Department, Ospedali Riuniti di Ancona, 60126 Ancona, Italy; 11 Cardiology Department, San Bortolo Hospital, 36100 Vicenza, Italy; 12 Medtronic Core Clinical Solutions, 00165 Rome, Italy; 13 Abbott EMEA Medical Affairs, 1931 Brussels, Belgium; 14 Cardiology Division, Department of Biomedical, Metabolic and Neural Sciences, Modena University Hospital, University of Modena and Reggio Emilia, 41125 Modena, Italy; 15 Clinical and Experimental Medicine PhD Program, University of Modena and Reggio Emilia, 41125 Modena, Italy

**Keywords:** atrioventricular conduction, death, heart failure, cardiac resynchronization therapy, pacing, programming

## Abstract

**Background.** Cardiac resynchronization therapy (CRT) is a treatment for heart failure (HF) patients with prolonged QRS and impaired left ventricular (LV) systolic function. We aim to evaluate how the baseline PR interval is associated with outcomes (all-cause death or HF hospitalizations) and LV reverse remodeling (>15% relative reduction in LV end-systolic volume). **Methods.** Among 2224 patients with CRT defibrillators, 1718 (77.2%) had a device programmed at out-of-the-box settings (sensed AV delay: 100 ms and paced AV delay: 130 ms). **Results.** In this cohort of 1718 patients (78.7% men, mean age 66 years, 71.6% in NYHA class III/IV, LVEF = 27 ± 6%), echocardiographic assessment at 6-month follow-up showed that LV reverse remodeling was not constant as a function of the PR interval; in detail, it occurred in 56.4% of all patients but was more frequent (76.6%) in patients with a PR interval of 160 ms. In a median follow-up of 20 months, the endpoint of death or HF hospitalizations occurred in 304/1718 (17.7%) patients; in the multivariable regression analysis it was significantly less frequent when the PR interval was between 150 and 170 ms (hazard ratio = 0.79, 95% confidence interval (CI): 0.63–0.99, *p* = 0.046). The same PR range was associated with higher probability of CRT response (odds ratio = 2.51, 95% CI: 1.41–4.47, *p* = 0.002). **Conclusions.** In a large population of CRT patients, with fixed AV pacing delays, specific PR intervals are associated with significant benefits in terms of LV reverse remodeling and lower morbidity. These observational data suggest the importance of optimizing pacing programming as a function of the PR interval to maximize CRT response and patient outcome.

## 1. Introduction

Cardiac resynchronization therapy (CRT) is an established treatment for patients with mild to severe heart failure (HF), impaired left ventricular (LV) systolic function, and prolonged QRS duration [1,2]. In most of these patients, CRT improves patients’ symptoms, induces LV reverse remodeling, and reduces all-cause mortality [3,4,5,6]. CRT response, based on LV reverse remodeling, ranges between 52% and 74% [7]; these values mainly pertain to patients with left bundle branch block (LBBB) who form about 70–90% of patients in CRT studies [8], while data on CRT-induced LV reverse remodeling in right bundle branch block (RBBB) is very limited [9,10]. Secondary analyses of landmark trials, such as the Comparison of Medical Therapy, Pacing, and Defibrillation in Heart Failure (COMPANION) [11] and Multicenter Automatic Defibrillator Implantation Trial with Cardiac Resynchronization Therapy (MADIT-CRT) [12,13] have recently suggested the importance of a baseline PR interval. In particular, Olshanski et al. [11] found that a PR interval longer than or equal to 200 ms was associated with a higher risk of all-cause mortality or HF hospitalization in those randomized to optimal pharmacological therapy but not in those randomized to CRT, suggesting that CRT may remedy the adverse effects of interatrial or interventricular conduction delays. Zareba et al. in the MADIT CRT trial [12] showed that CRT, compared with ICD-only therapy, resulted in LV reverse remodeling in all patients, regardless of QRS morphology, but that the risk of HF events or death was reduced only in left bundle branch block (LBBB) patients, not in right bundle branch block (RBBB) or other non-LBBB patients. Importantly, in the same population, Kutyifa et al. [13] showed that CRT may reduce the risk of death or HF events compared with ICD-only, even in non-LBBB patients if they had a prolonged PR interval. These data, all together, support the notion that the PR interval is a marker of atrial or ventricular conduction delays which worsen HF patients’ prognoses and that CRT may negate the deleterious effects of these conduction disturbances by improving interventricular synchrony and/or atrial–ventricular (AV) synchrony. This mechanistic conclusion was tested in several studies [14], by comparing CRT effects across different AV pacing delays, but results were not conclusive because those studies were single-center experiences, on small patient cohorts, and only observing acute echocardiographic endpoints. As a consequence, echocardiographic optimization is not indicated at present [1,2] and most devices are programmed using out-of-the-box settings.

In this context, we designed a retrospective analysis of data from consecutive CRT patients, included and followed in prospective projects, to evaluate the hypothesis that CRT response and long-term clinical outcomes depend on the baseline PR interval and specifically on the relationship between the PR interval and AV pacing delay.

## 2. Materials and Methods

### 2.1. Project Framework

In total, 100 Italian cardiology centers included consecutive patients wearing CRT defibrillators (CRT-D) in two prospective clinical projects, the Advance III trial (ClinicalTrials.gov identifier: NCT00617175) and the One Hospital ClinicalService, a medical care project targeting quality improvement in the use of Medtronic cardiac electronic implantable devices (CIED) in clinical practice. Data collection and analysis were approved by each participating site’s Institutional Review Board and complies with the Declaration of Helsinki. Each patient provided signed informed consent.

### 2.2. Patient Population

Patients were eligible for the analysis if they were implanted with a CRT-D according to international guidelines [1,2], i.e., systolic HF in New York Heart Association (NYHA) class III or ambulatory IV, or II in the case of a recent HF hospitalization, left ventricle ejection fraction (LVEF) ≤ 35%, and QRS ≥ 120 ms, despite maximum tolerated pharmacologic therapy. The inclusion period was from February 2013 to December 2018. Transvenous CRT-D implantation was undertaken using standard transvenous techniques under local anesthesia and targeting a lateral or posterolateral LV site for LV lead positioning. The used devices did not feature AV optimization algorithms.

### 2.3. Research Hypothesis, Methods, and Endpoints

We hypothesized that relevant improvements in LV reverse remodeling and clinical outcomes may be correlated to the baseline PR interval; we also aimed to evaluate if and how LBBB and RBBB mediate CRT response as a function of the baseline PR interval. The standard approach to evaluate CRT response through echocardiographic measurements was to evaluate the cardiac response in acute settings at different pacing AV delays in each patient [14]. We reversed that approach and evaluated the CRT response at 6 months of patients with different baseline PR intervals. With regard to the PR interval and device programming, after CRT-D implantation the clinicians set the AV pacing delay interval by maintaining the out-of-the-box setting (sensed AV (SAV) delay equal to 100 ms, paced AV (PAV) delay equal to 130 ms, and difference between the right ventricular and left ventricular (VV) pacing time equal to 0) in 1718 patients (77%), while in the remaining 506 patients (23%) different AV delays were programmed, according to each investigator’s usual clinical practice, including ECG or echocardiographic measurements. This is in line with the observational nature of this study that reflects daily practice [15]. At the follow-up visits, when CRT response was assessed, in all patients, who resulted as CRT non-responders, AV and VV delays were optimized by echocardiography according to each site clinical practice.

Baseline patients’ characteristics were collected before CRT-D implantation. Standard 12-lead ECGs were collected at patient enrollment and were analyzed by each site’s investigators. Ventricular conduction disturbances, in particular LBBB and RBBB conditions, were defined according to AHA/ACCF/HRS recommendations [16]. The PR interval was estimated as the time between P wave onset and QRS onset at ECG measurements recorded before CRT implant at a 50 mm/s paper speed to increase measurement accuracy. Transthoracic echocardiogram measurements, performed with the Simpson’s biplane method [17] at baseline and at the 6-month follow-up visit after implantation, provided information about LVEF and LV end-systolic volume (LVESV). Research endpoints comprised LV reverse remodeling (LVESV relative reduction > 15%), all-cause death, and HF hospitalizations and incidence of atrial fibrillation (AF) longer than 7 days, a duration chosen because it has been associated with persistent AF which is associated with worse outcomes [18]. Information about the occurrence and duration of AF after the implant was extracted from the device diagnostics. Information about life status and HF hospitalizations were collected at follow-up visits, scheduled according to each center’s clinical practice or through telephone contacts with patients or their relatives.

### 2.4. Statistical Analysis

All characteristics reported were described using summary statistics. Continuous variables were expressed as means and standard deviations or median and interquartile range (IQR), as appropriate. Categorical variables were expressed as counts and percentages. Rates were computed for 100 person years and were compared by means of the Poisson model using the scale deviation parameter to adjust for over-dispersion. Cox regression models were implemented in order to find independent predictors of clinical endpoints and CRT response. The backward selection method was used to manage inclusion and exclusion in the final multivariable models (backward selection method: in *p*-value = 0.05, out *p*-value = 0.05). Odds ratios and hazard ratios (HR) and 95% confidence interval (95%CI) were also calculated. No imputation of missing data was performed. The SAS software (SAS Institute Inc., Cary, NC, USA) was used to perform statistical analyses. Statistical tests were based on a two-sided significance level of 0.05.

## 3. Results

From an overall population of 2224 CRT-D patients we selected 1718 (77.2%) patients who had pacing delays programmed with out-of-the-box settings. These patients form the cohort analyzed and described in the following. In particular, 78.7% patients were men, with a mean age of 66 ± 10 years. All patients had advanced-heart-failure symptoms with most patients (71.6%) in NYHA functional class III or IV. The etiology of the underlying cardiomyopathy was ischemic in 53% of patients. Patients had severely depressed LV function and extensive LV dilation, as shown in Table 1. Medication included diuretics in 85.2% of patients, ACE-inhibitors or ARB in 81.4% of patients, and beta-blockers in 73.7%% of patients. Median paced QRS duration was 140 ms (IQR = 120–160 ms). In the overall population the median of ventricular pacing percentage, collected through device diagnostics every day, was 99% (IQR = 99%–99%).

### 3.1. LV Reverse Remodeling

At 6 months LVESV decreased from 154 ± 64 mL to 128 ± 64 mL (*p* < 0.001). Overall, the percentage of patients who were classified as responders to CRT according to LVESV >15% relative decrease was 56.4%. When considering QRS morphology, the percentage of CRT responders was 58.0% in LBBB and 52.6% in RBBB. LVEF also improved from baseline (27 ± 6%) to 6 months follow-up (34 ± 9% (*p* < 0.001), with 62% of patients who were classified as responders to CRT according to LVEF > 5% absolute increase.

### 3.2. LV Reverse Remodeling and PR Interval

**Figure 1:**
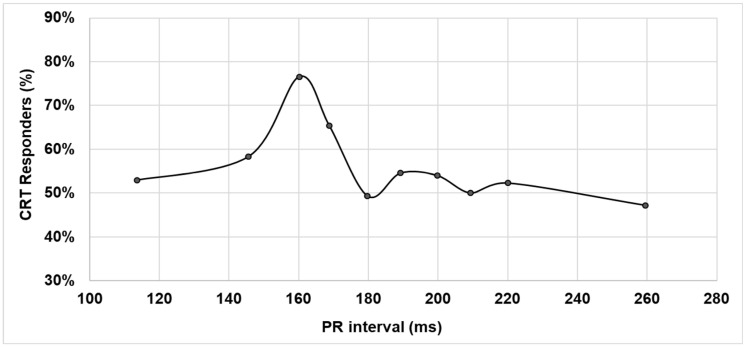
CRT response as a function of the PR interval in all patients.

The percentage of responders to CRT was not constant as a function of the baseline PR interval. Rather, CRT response in terms of LVESV reduction showed a sharp peak—higher than 76%—for the baseline PR interval equal to 160 ms, as shown in Figure 1.

**Figure 2:**
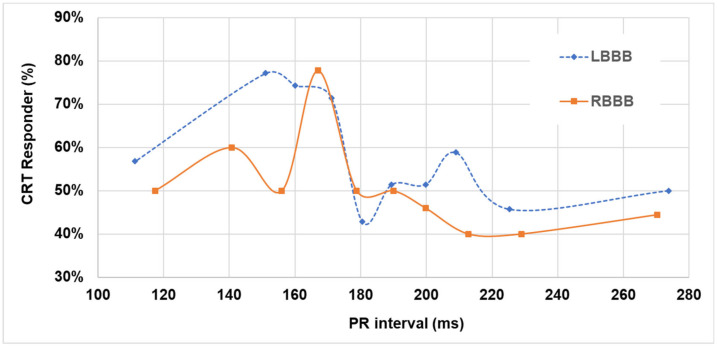
CRT response as a function of the PR interval according to QRS morphology.

Dividing the whole population into patients with LBBB and into patients with RBBB, a similar behavior was observed with a large peak—higher than 70%—at baseline PR intervals comprised between 150 and 170 ms for LBBB patients, and a sharp peak for the baseline PR interval at about 170 ms for RBBB patients, as shown in Figure 2. In the univariate and multivariable analyses, the probability of being responder to CRT, in terms of LVESV reduction (Table 2), was significantly associated with baseline PR intervals in the range between 150 and 170 ms (odds ratio = 2.51, 95%CI = 1.41–4.47, *p* = 0.002), and not to baseline QRS morphology. The median paced QRS duration was 130 ms (IQR = 120–150 ms) in patients with baseline PR intervals in the range between 150 and 170 ms and 150 ms (IQR 130–160 ms) in patients with a PR interval >170 ms (*p* < 0.001).

### 3.3. Clinical Outcomes

**Figure 3:**
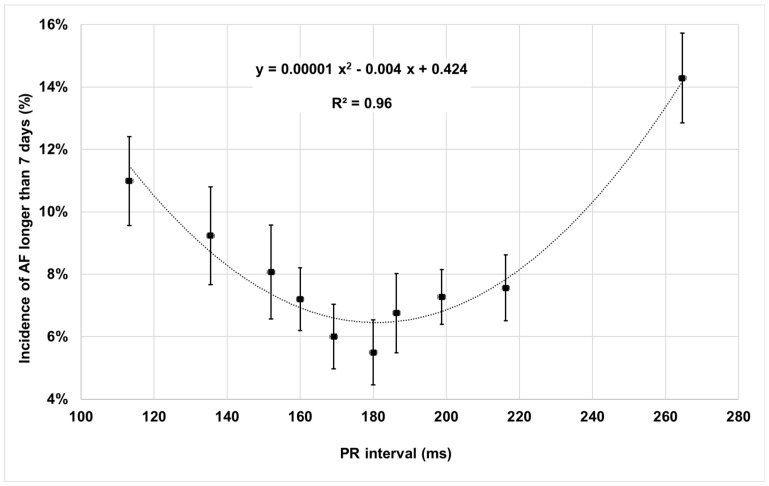
Incidence of atrial fibrillation longer than 7 days as a function of the PR interval.

During a median follow-up of 20 months (IQR = 12–42 months), AF longer than 7 days occurred in 149/1718 (8.7%) patients. The incidence of AF longer than 7 days as a function of the baseline PR interval is described in Figure 3. AF incidence data were fitted with a second-order polynomial function which showed a minimum AF incidence at about PR = 180 ms with a very high fit correlation (R square = 0.96).

All-cause death or HF hospitalizations occurred in 304/1718 (17.7%) patients. Several baseline characteristics were associated with all-cause death or HF hospitalizations (Table 3). While all the other variables, such as age > 65 years, secondary sudden death prevention, AF, and diabetes, were associated with a higher risk of the clinical composite endpoint, the baseline PR interval, when composed between 150 and 170 ms, was the only variable resulting as significantly and independently associated with a reduced risk of all-cause death or HF hospitalizations (HR = 0.79 (95%CI = 0.63–0.99), *p* = 0.046). By adding CRT response at 6 months to the multivariable analysis we found that previous predictors were confirmed and that an LVESV relative decrease > 15% was associated with a reduced risk of all-cause death or HF hospitalizations (HR = 0.40 (95%CI = 0.24–0.66), *p* < 0.001).

## 4. Discussion

Despite refinements in CRT indication and improvements in CRT technologies, still today a relevant proportion (26–48%) of patients do not respond to CRT [7]. Research on CRT response is mainly focusing on the electrophysiological mechanisms behind CRT benefit [19] and on patients’ characteristics, such as QRS duration and morphology [1,2,8,12] and/or baseline PR interval duration [11,13]. Additionally, pacing site and multisite pacing [20] and AV optimization algorithms [21,22,23,24] are currently evaluated to improve CRT response.

The PR interval, AV pacing delays, and bundle branch block characteristics interplay, determining interventricular synchrony and AV synchrony. We therefore hypothesized that an observation of reverse remodeling and clinical outcomes as a function of the baseline PR interval and QRS morphology, at a fixed out-of-the-box pacing AV delay, would have spread light on CRT response.

Our data show that about 77% of CRT devices in clinical practice are programmed with out-of-the-box AV delay programming. Indeed, many implanters do not use either echocardiographic or ECG methods to optimize the AV interval but instead empirically program devices to a fixed AV delay interval and optimize only those patients who fail to respond to therapy [21]. This situation is likely to persist until new AV delay optimization algorithms [21,22,23,24] show significant clinical benefits. Our analyses demonstrate that simultaneous biventricular pacing delivered with a sensed AV delay equal to 100 ms results in a high CRT response—over 76%—for patients with a baseline PR interval between 150 and 170 ms. This phenomenon of high CRT response at very specific baseline PR intervals was observed in both LBBB and RBBB patients. As expected, in the univariate analysis, the risk of death or HF hospitalizations was significantly lower in patients with LBBB and significantly higher in patients with a PR interval > 200 ms. The novelty of our analysis was to measure the impact of the PR interval, not only considering PR interval as a dichotomized variable, as normal or prolonged, but especially considering it as a continuous variable. When considering the PR interval, according to 10 discrete categories, a baseline PR interval in the range of 150–170 ms resulted as independently and significantly associated with improved prognosis.

### 4.1. LV Reverse Remodeling as a Function of PR Interval

Our data show that, overall, 56.4% of patients were responders to CRT. This raw percentage, averaged on all PR intervals, is aligned with those measured by previous studies [1,2,3,4,5,6,7]. Importantly our data show that LV reverse remodeling was not constant as a function of the baseline PR interval, rather CRT response showed a peak at 76.6% for the baseline PR interval near 160 ms (Figure 1). This percentage of CRT responders is higher than in other CRT studies; indeed, in a literature review [7] performed on 22 major CRT clinical trials the CRT response, based on LV reverse remodeling, ranged between 52% and 74%. The observation that fixed AV and VV pacing delays—SAV = 100 ms, PAV = 130 ms, VV = 0 ms—provide optimal VV and AV timing for patients with baseline PR intervals in the range 150–170 ms may suggest that patients with specific PR values would benefit from specific AV/VV pacing times, outlining the importance of AV optimization tailored to each patient’s conditions. An alternative hypothesis could be that patients with a baseline PR interval in the range of 150–170 ms, which is the physiologic and more frequent PR range, may identify a group of less-compromised patients who obtain a higher benefit from CRT.

### 4.2. LV Reverse Remodeling as a Function of Baseline PR Interval and QRS Morphology

The characterization of the CRT response as a function of the baseline PR interval in LBBB and in RBBB patients (Figure 2) provides interesting insights on these patient subgroups. Indeed, while all CRT trials [1,2,3,4,5,6] have included patients according to QRS duration, and therefore both LBBB and RBBB patients, secondary analyses of those trials have shown higher benefit in LBBB patients, and therefore guidelines (1–2) indicate CRT at class I, level of evidence A, in LBBB patients. Conversely, data on CRT response in RBBB is very limited [8,9,10]. Pastore et al. [9] found a high variability of CRT responders (according to LVESV 15% reduction), ranging between 19.4% and 71.4% according to RBBB type, RBBB being atypical and typical, respectively. Tompkins et al. [10] found that the mean percent reduction in LVESV between baseline and 12-month follow-up visits ranges between 21 and 28% according to presence or absence of a concomitant left anterior fascicular block.

In LBBB patients the window of PR intervals, which benefit from a simultaneous biventricular pacing with sensed AV delay at 100 ms, is large, comprised between 150 and 170 ms (Figure 2) possibly due to fusion between spontaneous RV and paced LV depolarizations. Fusion would take place when the AV delay is about 50–60 ms shorter than the PR interval, in other words, programmed at a value in the range of 62–67% of the PR interval. In RBBB patients, for whom there is no spontaneous RV depolarization, fusion would be possible only between right and left paced depolarizations and only at very specific timing conditions; this would explain the sharp peak in CRT responders as shown in Figure 2. The observation that CRT response rapidly decreases for patients with short or long PR intervals, compared with the PR interval range of 150–170 ms, is consistent with the hypothesis of loss of VV and/or AV synchrony. For example, in LBBB patients with short PR intervals, say, for example, PR ≤ 120 ms—which may occur in about 6% of patients—setting an AV delay at 100 ms results in no LV pre-excitation or in other words that the LV becomes activated by the spontaneous RV depolarization through the septum, therefore, there is no modification of LBBB; while in patients with long PR intervals, for example, PR ≥ 200 ms—which may occur in up to 44% of patients—setting a AV delay at 100 ms may result in too early LV activation with negative hemodynamic consequences due to too short left AV intervals [25], which may lead to reduced diastolic filling times, reduced atrial contribution to LV stroke volume, high left atrial pressure, mitral regurgitation, and ultimately to a condition similar to pacemaker syndrome [26]. In outliers of the optimal PR interval range, AV and VV tailoring rather than shipment settings may prove beneficial, especially when dynamically tailoring occurs according to changes in the PR interval [24,27].

### 4.3. Clinical Outcomes as a Function of Baseline PR Interval

The incidence of AF longer than 7 days showed a U-shape as a function of the baseline PR interval with a minimum at PR = 180 ms and higher values for shorter or longer PR intervals (Figure 3). This finding is consistent with the results of the Adaptive CRT trial (27) which recently showed higher AF incidence when echo-optimized biventricular pacing was delivered with too short or too long AV delays, i.e., too early or too late stimulation, which may cause reduced time for atrial contribution to ventricular filling and hemodynamic derangements due to asynchronous mitral valve closure. The risk of the endpoint composed by all-cause mortality and HF hospitalizations was reduced when the PR interval was in the range 150–170 ms, as shown in Table 3. This result is consistent with the fact that CRT response is higher within the same PR interval ranges (Table 2) and with the knowledge that when CRT induces LV reverse remodeling then patients have lower risk of death or HF events [28]. The important role of the PR interval, suggested by our data, has been recently described by Rickard et al. [29] who showed that, among LBBB patients, PR interval is an important predictor together with QRS duration in assessing long-term outcomes. Those authors have also found differences in the association between a long PR interval and worse clinical outcomes comparing LBBB patients with non-LBBB patients and have suggested an interplay of PR interval and CRT programming according to the specific conduction disturbances.

### 4.4. Clinical Implications

By considering the PR interval in its continuum of values, our research has unveiled the interplay between the PR interval and AV pacing delay programming. Higher CRT response and better clinical outcomes at specific PR intervals, when using fixed AV delays, suggest that clinical benefits may derive from optimizing AV pacing time according to patients’ PR intervals. These results confirm the electrophysiological mechanisms behind algorithms devoted to optimizing synchronicity among biventricular pacing and right ventricular spontaneous depolarization [27,29,30]. Our data show a high CRT response rate also in RBBB patients with specific PR intervals. This finding may have relevant clinical implications because patients with RBBB QRS morphology represent a sizeable subgroup of patients indicated to CRT [8] but so far, the benefit of biventricular pacing in patients with RBBB has been controversial [1,2,8,12] and, as a consequence, International Guidelines [1,2] indicate CRT in non-LBBB patients only at class IIa pr IIb according to QRS duration.

### 4.5. Study Limitations

Our research has some limitations, such as the retrospective nature of our analyses, even if data collection was prospective. Our results do apply to the subgroup of patients with out-of-the-box settings with fixed AV and VV delays; also, our results apply to a population of patients treated in the period 2013–2018 and it is possible that response to CRT in contemporary practice may be better thanks to the progressive use of quadripolar leads and AV optimization algorithms. We analyzed the occurrence of AF > 7 days as a function of PR interval, even if many factors may actually condition AF onset in patients with LV dysfunction and AF [2,27,31,32,33,34,35]. Additionally, given the observational nature of the study, granular descriptions of specific echocardiographic measurements, such as grade and type of valvular heart disease, were missing. Alongside these limitations, we believe our project had solid methods, such as a pre-specified statistical plan, and has strengths deriving from the fact that it included a large number of CRT patients, who were consecutively included and prospectively followed by 100 cardiology centers in their real-world clinical practice, accurately collecting hard endpoints, such as death and HF hospitalizations, device-derived AF incidence, and burden and echocardiographic measurements.

## 5. Conclusions

In a large multicenter prospective analysis, most (77%) CRT devices are left at out-of-the-box settings. This condition allowed us to perform a fine continuous scanning of CRT response according to baseline PR interval values and at fixed AV and VV delay settings. Our data show that CRT-induced reverse remodeling is not constant as a function of the PR interval, rather the percentage of CRT response is characterized by a sharp peak, at 76%, associated with a PR interval between 150 and 170 ms. This finding was observed, with slight differences, both in LBBB and RBBB patients. This range of PR intervals was significantly associated with reduced incidence of all-cause death or HF hospitalizations. Overall, these results suggest that it may be clinically important to optimize the synchronicity between atrial and ventricular depolarizations and between biventricular pacing and ventricular spontaneous depolarizations.

## Figures and Tables

**Table 1:** Patient demographics and baseline characteristics.

Analyzed Cohort (1718 Patients)	
*Patient demographics*	
Age (years) mean ± SD	66 ± 10
Gender (male) (%)	78.7%
*Medical history*	
Secondary prevention (%)	12.4%
Atrial tachyarrhythmias (%)	21.9%
Ischemic cardiopathy (%)	53.0%
Myocardium infarction (%)	47.0%
Right bundle branch block (%)	17.6%
Left bundle branch block (%)	70.8%
New York Heart Association Classes III–IV (%)	71.6%
Hypertension (%)	57.9%
Diabetes (%)	28.8%
History of stroke/TIA (%)	3.7%
Valvular disease (%)	21.1%
*ECG and echo measures*	
QRS duration (ms), mean ± SD	151 ± 29
PR interval (ms), median (IQR)	182 (160–204)
P wave duration (ms), median (IQR)	80 (60–90)
Left ventricle ejection fraction (%), mean ± SD	27 ± 6
Left ventricle end-diastolic volume (ml), mean ± SD	209 ± 78
Left ventricle end-systolic volume (ml), mean ± SD	154 ± 64
Left ventricle end-diastolic diameter (mm), mean ± SD	70 ± 40
Left ventricle end-systolic diameter (mm), mean ± SD	60 ± 39
Left atrium diameter (mm), mean ± SD	49 ± 16
*Baseline medical therapy*	
Beta-blocker (%)	73.7%
Diuretic (%)	85.2%
Antiarrhythmics (%)	21.3%
ACE-inhibitor/ARB (%)	81.4%
Digitalis (%)	15.8%

Legend: ACE = angiotensin-converting enzyme; ARB = angiotensin-receptor blockers; SD = standard deviation.

**Table 2:** Univariate and multivariate analysis of predictors of CRT response.

	Univariate		Multivariate	
	***OR (95% CI)***	***p Value***	***OR (95% CI)***	***p Value***
PR > 200 ms	**0.68 (0.46–0.98)**	**0.040**		
150 ms ≤ PR ≤ 170 ms	**2.17 (1.46–3.23)**	**<0.001**	**2.51 (1.41–4.47)**	**0.002**
Age > 65 years	0.82 (0.59–1.15)	0.243		
Gender (male)	**0.64 (0.42–0.97)**	**0.034**		
Secondary prevention	**0.57 (0.34–0.98)**	**0.042**		
Atrial tachyarrhythmias	0.70 (0.47–1.05)	0.088		
Hypertension	1.41 (0.93–2.16)	0.107		
Diabetes	0.66 (0.42–1.04)	0.072	**0.37 (0.20–0.67)**	**0.001**
History of stroke/TIA	0.58 (0.19–1.75)	0.329		
Valvular disease	0.69 (0.47–1.00)	0.053	**0.52 (0.29–0.93)**	**0.027**
Ischemic cardiomyopathy	**0.48 (0.34–0.67)**	**<0.001**	**0.52 (0.30–0.89)**	**0.018**
Myocardium infarction	**0.52 (0.37–0.75)**	**<0.001**		
RBBB	0.85 (0.55–1.32)	0.477		
LBBB	1.26 (0.89–1.77)	0.193		
NYHA 3/4	0.76 (0.52–1.11)	0.162		
QRS > 150 ms	1.28 (0.92–1.78)	0.147		
LVEF ≤ 25%	0.88 (0.63–1.23)	0.464		
ACE-inhibitor/ARB	1.13 (0.71–1.78)	0.614		
Beta-blocker	1.34 (0.91–1.96)	0.139		
Digitalis	0.95 (0.59–1.54)	0.846		
Diuretics	0.79 (0.48–1.31)	0.365		
Antiarrhythmics	0.76 (0.51–1.13)	0.178		

Legend: OR = odds ratio; TIA = transient ischemic attack; RBBB = right bundle branch block; LBBB = left bundle branch block; NYHA = New York Heart Association; LVEF = left ventricle ejection fraction; ACE = angiotensin-converting-enzyme; ARB = angiotensin-receptor blockers.

**Table 3:** Univariate and multivariate Cox regression analysis of the risk of all-cause death or HF hospitalization.

	Univariate		Multivariate	
	***HR (95% CI)***	***p Value***	***HR (95% CI)***	***p Value***
**PR > 200 ms**	**1.50 (1.21–1.85)**	**<0.001**		
**150 ms ≤ PR ≤ 170 ms**	**0.74 (0.59–0.93)**	**0.011**	**0.79 (0.63–0.99)**	**0.046**
**Age > 65 yrs**	**1.57 (1.27–1.94)**	**<0.001**	**1.26 (1.00–1.59)**	**0.050**
**Gender (male)**	**1.49 (1.14–1.94)**	**0.004**		
**Secondary prevention**	**1.62 (1.26–2.09)**	**<0.001**	**1.37 (1.03–1.84)**	**0.032**
**Atrial tachyarrhythmias**	**1.72 (1.38–2.13)**	**<0.001**	**1.64 (1.30–2.08)**	**<0.001**
Hypertension	1.11 (0.88–1.41)	0.365		
**Diabetes**	**1.98 (1.58–2.48)**	**<0.001**	**2.15 (1.49–3.10)**	**<0.001**
History of stroke/TIA	1.34 (0.82–2.19)	0.236		
Valvular disease	1.13 (0.89–1.44)	0.316		
**Ischemic cardiomyopathy**	**1.64 (1.33–2.01)**	**<0.001**	**1.45 (1.15–1.83)**	**0.002**
**Myocardial infarction**	**1.67 (1.36–2.05)**	**<0.001**		
RBBB	1.23 (0.91–1.66)	0.175		
**LBBB**	**0.80 (0.65–0.99)**	**0.041**		
**NYHA Class III or IV**	**1.51 (1.20–1.91)**	**<0.001**		
QRS > 150 ms	0.91 (0.75–1.11)	0.361		
**EF ≤ 25%**	**1.25 (1.01–1.53)**	**0.037**		
ACE-inhibitor/ARB	0.82 (0.64–1.05)	0.117		
**Beta-blocker**	**0.56 (0.46–0.70)**	**<0.001**		
Digitalis	1.23 (0.93–1.62)	0.141		
**Diuretics**	**1.44 (1.06–1.97)**	**0.019**		
**Antiarrhythmics**	**1.57 (1.26–1.95)**	**<0.001**		

Legend: HR = hazard ratio; TIA = transient ischemic attack; RBBB = right bundle branch block; LBBB = left bundle branch block; NYHA = New York Heart Association; LVEF = left ventricle ejection fraction; ACE = angiotensin-converting enzyme; ARB = angiotensin-receptor blockers.

## Data Availability

The data presented in this study are not publicly available. Restrictions may apply to the availability of these data due to the fact that data were owned by contributing sites.

## Appendix A. Research Participating Centers

Patients included in the presented analysis were included and followed within clinical projects (ClinicalTrials.gov identifiers: NCT00617175).

IRCCS Humanitas Research Hospital—Rozzano; Ospedale Niguarda Ca Granda—Milano; Policlinico San Matteo—Pavia; Ospedale Civile Maggiore di Borgo Trento –Verona; Az. Ospedaliera S.Maria della Misericordia—Udine; Ospedale Mater Salutis di Legnago—Legnago; Ospedale San Filippo Neri—Roma; Ospedale Santa Maria Del Carmine—Rovereto; A.S. Ospedaliera S. Croce e Carle—Cuneo; Ospedale San Raffaele—Milano; Villa S. Anna S.p.A.—Catanzaro; Azienda Ospedaliera Sacro Cuore Don Calabria—Negrar; Ist. Auxologico Italiano-Ospedale S.Luca—Milano; Ospedale Civile G. Mazzini—Teramo; Osp. S. Maria degli Angeli—Pordenone; Policlinico Sant Orsola-Malpighi—Bologna; Ente Ospedaliero Ospedali Galliera—Genova; Ospedale Santa Maria della Misericordia—Rovigo; Azienda Ospedaliera Careggi—Firenze; Ospedale SS. Antonio e Biagio—Alessandria; ULSS N.6 S. Bortolo—Vicenza; Ospedale San Carlo Borromeo—Milano; Ospedale Humanitas Gavazzeni—Bergamo; Ospedale Luigi Sacco—Milano; Ospedale San Gerardo—Monza; Ospedale S.Anna—Como; Ospedale Belcolle—Viterbo; Ospedale Loreto Mare—Napoli; AZ. Osp. Ordine Mauriziano—Torino; Osp. S. Giovanni di Dio e Ruggi d’Aragona—Salerno; Azienda Ospedaliera Pugliese e Ciaccio—Catanzaro; Ospedale Civile dello Spirito Santo—Pescara; Azienza Ospedaliera Spedali Civili—Brescia; Az. Osp. Molinette S.Giovanni Battista—Torino; Ospedale Vito Fazzi—Lecce; Ospedale Sandro Pertini—Roma; Presidio ospedaliero C.e G. Mazzoni -Ascoli Piceno; Ospedale Misericordia e Dolce—Prato; Ospedale Civile di Conegliano—Conegliano Veneto; Ospedale Civile—Asti; Ospedali Riuniti—Bergamo; Ospedale Sant Eugenio—Roma; Ospedale Civile G. Fornaroli—Magenta; Osp. San Giovanni Calibita Fatebenefratelli—Roma; A.O. Carlo Poma—Pieve di Coriano; Ospedale Regionale San Maurizio—Bolzano; Azienda Ospedaliera San Salvatore—Pesaro; Az. Osp. Ca Foncello—Treviso; Ospedale S. Paolo P.M.—Milano; Azienda Ospedaliera Vittorio Emanuele Ferrarotto—S. Bambino—Catania; Ospedale Ferrarotto—Catania; Ospedale di Circolo—Desio; Ospedale Fatebenefratelli e Oftalmico—Milano; Presidio Ospedaliero Riunito—Ciriè; P.O. di Montebelluna—Montebelluna; Nuovo Osp. Civile S. Agostino—Estense—Modena; Osp. S. Orsola-Fatebenefratelli—Brescia; Ospedale P. Cosma—Camposampiero; Casa di Cura Mater Domini—Castellanza; Ospedale di Circolo—Busto Arsizio; Ospedale Civile di Mirano—Mirano; Ospedale Maggiore—Lodi; Ospedale SS. Annunziata—Chieti; Ospedale Giovan Battista Grassi—Ostia; Stab. Ospedaliero Di Summa-Perrino—Brindisi; Ospedale Civile—Legnano; Centro Cuore Morgagni—Pedara; Facoltà di Medicina e Chirurgia—Bari; Ospedale Bianchi-Melacrino-Morelli—Reggio Calabria; Ospedale Civile—Arzignano; Casa di Salute Montevergine—Mercogliano; Ospedale L. P. Delfino ASL Roma G—Colleferro; Fond. Ist. S.Raffaele-G. Giglio—Cefalù; Policlinico Casilino—Roma; Ospedali Riuniti di Ancona—Ancona; Azienda Ospedaliera di Padova—Padova; Ospedale S. Giacomo—Novi Ligure; Azienda Ospedaliera Maggiore della Carità—Novara; Ospedale di Manerbio-Leno—Manerbio; Ospedale S.Pietro Igneo—Fucecchio; S. Maria Nuova Hospital—Reggio Emilia; Ospedale S. Donato—San Donato Milanese; Azienda Ospedaliera Carlo Poma—Mantova; Ospedale di Desenzano—Desenzano del Garda; Ospedale Maggiore—Policlinico—Milano; Ospedale Civile di Vimercate—Vimercate; Presidio Ospedaliero di Milazzo (AUSL 5)—Milazzo; Ospedale S. Maria delle Croci—Ravenna; Ospedale G. Moscati—Avellino; Azienda Ospedaliera Bolognini—Seriate; Fondazione Poliambulanza—Brescia; Azienda Ospedaliera G. Rummo—Benevento; Ospedale S. Spirito—Casale Monferrato—Alessandria; Ospedale SS. Giacomo e Cristoforo—Massa; Osp. Santa Maria delle Grazie—Pozzuoli (NA); Ospedale San Vincenzo—Taormina; Casa di Cura Villa Verde—Taranto; A.U.S.L.—Osp. Guglielmo da Saliceto di Piacenza; Ospedale Oglio Po—Vicomoscano di Casalmaggiore; Presidio Ospedaliero di Venere—Bari Carbonara; Az. ULSS 12 Veneziana—Osp. Dell Angelo—Mestre.
